# Personal infection prevention behaviors and campaign to encourage travel during COVID-19: A cross-sectional study

**DOI:** 10.3389/fpubh.2023.1037496

**Published:** 2023-02-01

**Authors:** Hayato Shimoda, Tomohisa Nagata, Tomohiro Ishimaru, Ayako Hino, Hajime Ando, Keiji Muramatsu, Seiichiro Tateishi, Mayumi Tsuji, Koji Mori

**Affiliations:** ^1^Department of Occupational Health Practice and Management, Institute of Industrial Ecological Sciences, University of Occupational and Environmental Health, Kitakyushu, Japan; ^2^Department of Environmental Epidemiology, Institute of Industrial Ecological Sciences, University of Occupational and Environmental Health, Kitakyushu, Japan; ^3^Department of Mental Health, Institute of Industrial Ecological Sciences, University of Occupational and Environmental Health, Kitakyushu, Japan; ^4^Department of Work Systems and Health, Institute of Industrial Ecological Sciences, University of Occupational and Environmental Health, Kitakyushu, Japan; ^5^Department of Preventive Medicine and Community Health, School of Medicine, University of Occupational and Environmental Health, Kitakyushu, Japan; ^6^Disaster Occupational Health Center, Institute of Industrial Ecological Sciences, University of Occupational and Environmental Health, Kitakyushu, Japan; ^7^Department of Environmental Health, School of Medicine, University of Occupational and Environmental Health, Kitakyushu, Japan

**Keywords:** Go To Travel, infection prevention behaviors, cross-sectional study, COVID-19, Japan

## Abstract

**Introduction:**

The Go To Travel campaign in Japan was launched to subsidize travel and accommodation costs for tourists through vouchers that could be used at domestic destinations. Infection prevention behavior can lead to refraining from travel owing to infection concerns; conversely, taking preventive action can promote travel. There is a lack of information about the association between infection prevention behaviors and desire to travel. During a pandemic of infection, there is the difficult challenge of balancing the promotion of infection prevention behavior with economic revitalization. Thus, we examined the relationship between personal infection prevention behaviors and using Go To Travel.

**Methods:**

We conducted a cross-sectional study of 26,637 workers who responded to a large-scale questionnaire survey about COVID-19 in Japan. We built multilevel logistic regression models adjusted for confounders to assess the association between each personal infection prevention behavior and using Go To Travel. We analyzed the seven infection prevention behavior individually: wearing a mask when among other people; disinfecting hands with alcohol before going indoors; washing hands after using the toilet; gargling upon returning home; opening a window to ventilate a room; carrying an alcohol sanitizer; and disinfecting hands after touching objects outside.

**Results:**

Among the 26,637 participants, 7,959 (30%) used Go To Travel. For “wearing a mask in the presence of others” and “washing hands after using the toilet,” the majority of respondents answered “almost always: 86.5 and 85.6% respectively. Action “carrying alcohol disinfectant” was the least implemented, with 36.9% of respondents saying “almost never.” Two of the seven preventive behaviors (“disinfecting hands with alcohol before going indoors” and “carrying alcohol disinfectant”) were positively related to using Go To Travel, that is, the more of these actions they took, the more they used Go To Travel (*p* for trend <0.001).

**Conclusions:**

To balance pandemic preparedness with economic preparedness, it is also necessary to promote travel when the infection situation is calm. However, since human mobility can be a factor that exacerbates the infection situation, it is necessary to promote more infection prevention behaviors among individuals. We confirmed that Go To Travel users were basically doing a good infection prevention behaviors, but they tended not to wash their hands after touching things or carry alcohol sanitizer. It is necessary to encourage these measures to be taken when traveling.

## 1. Introduction

As COVID-19 control measures, many countries have implemented lockdowns and other activity restrictions. Such measures have had a significant impact on health and on the economy (1). In Japan, four emergency declarations were made by the government before the end of 2021: they called on the public to refrain from going out and requested restrictions on restaurants and other places of entertainment (2). To reduce the economic impact, the Japanese government implemented various support measures (3–5). The government introduced a subsidy program to support the tourism, and food and beverage industries (which were particularly affected economically) (3). The program comprised Go To Travel and Go To Eat. Among them, launched on July 22, 2020, the Go To Travel campaign focused on subsidizing travel and accommodation costs for tourists and issuing vouchers that could be used at domestic destinations (3). The subsequent reemergence of COVID-19 forced the government to suspend that campaign on December 28, 2020; it declared a second state of emergency in some prefectures on January 7, 2021 (4, 6).

Concerns were expressed that the Go To Travel would lead to increased mobility, which could spread infection (7–9). The mobility, connectivity, and availability of traffic resources can be crucial of virus-spreading paths (10). While a study suggest that Japan's Go To Travel campaign may have caused the spread of infection from urban to rural areas (11), the other suggest that go-to-travel has no lasting impact on the spread of infection (12), and findings are not constant. However, in theory, mobility itself is a small factor in spreading infection: appropriate infection prevention behaviors during travel and at travel destinations could prevent infection spread (13). There has, though, been no study about infection prevention behaviors among Go To Travel users. Owing to this lack of information, infection prevention behaviors when traveling deserve analysis. An indirect way to examine that relationship would be to assume that infection prevention behaviors when traveling correlate with routine infection prevention behaviors and to examine the relationship between routine behaviors and using Go To Travel. We hypothesized that individuals who adopt infection prevention behaviors use Go To Travel more extensively.

The relationship between infection prevention and outdoor behaviors is likely influenced by the balance between those potentially positive and negative factors. It has been found that compliance with infection prevention behaviors is influenced by the risk perception of infection—specifically, perceived severity, susceptibility, self-efficacy of infection prevention behavior, and effectiveness of measures (14–16). Confidence in governmental programs has been reported to be positively correlated with infection prevention behaviors (15). These findings would result in inhibitions about traveling. Conversely, perceived self-efficacy and effectiveness may lead to an awareness of the effectiveness of infection prevention behaviors when traveling: they could be an active influence on traveling. Confidence in the Japanese government is also considered a positive factor in accepting Go To Travel (a government-led campaign). We also hypothesized that those who are more risk-conscious (such as people who are more anxious about infection) are more likely to take infection-prevention measures and be reluctant to use Go To Travel.

We examined the relationship between infection prevention behaviors and using Go To Travel; we also investigated how fear of COVID-19 infection affected that relationship.

## 2. Materials and methods

This is a cross-sectional study conducted online on December 22-25, 2020. The 605,381 people of Internet monitors were emailed invitations to ascertain whether or not they were willing to participate. Subsequently, those who met the inclusion criteria (worker status, region, gender, and age) for this study were selected. Details of the sampling method and other details have already been reported in the previous paper (17) and shown in Supplementary Table 1 (18).

We excluded individuals who did not respond adequately. We also excluded individuals who had been infected with COVID-19 and those who had been identified as having been in close contact with infected people because they were inappropriate for our study purposes.

This study was approved by the ethics committee of the University of Occupational and Environmental Health, Japan (reference No. R2-079 and R3-006).

### 2.1. Explanatory variable and outcomes

We asked participants about the following seven infection prevention actions with four options (almost always, often, not often, or almost never): “In the last month, have you taken any of the following actions?”: wearing a mask when among other people; disinfecting hands with alcohol before going indoors; washing hands after using the toilet; gargling upon returning home; opening a window to ventilate a room; carrying an alcohol sanitizer; and disinfecting hands after touching objects outside. These seven items were based on the infection control guidelines for COVID-19 drawn up by the WHO and the Japanese government for the general public (19–22). We defined “almost always” or “often” as having good behavior and counted the number of such good behaviors.

We asked participants about using Go To Travel with the question, “Did you use Go To Travel?” There were two options—yes or no.

We also asked participants about their fear about COVID-19 infection with the question, “Do you feel anxious about COVID-19 infection?” There were two options—yes or no.

### 2.2. Covariates

The covariates comprised sociodemographic factors, socioeconomic factors, and occupation. We chose those covariates because previous studies have indicated that the following characteristics are associated with infection prevention behavior: male (14, 23–25); younger (23–25); lower income (14, 24); lower education (25); anxiety (14, 25); and rural residence (14, 24). Marital status and occupation are considered factors related to willingness to undertake travel. These factors were therefore considered confounding factors and added to the covariates.

We classified age into five categories: 20–29, 30–39, 40–49, 50–59, and 60–65 years. We divided annual household income into six groups: under 2 million, 2–4 million, 4–6 million, 6–8 million, 8–10 million, and over 10 million yen. Education was categorized into three groups: junior high school or high school graduates; vocational school, junior college, or technical college graduates; and university or graduate school graduates. We classified marital status into three categories: married, widowed or divorced, and never married. Occupations were divided into 10 groups: general employee; manager; executive; public employee, faculty member, or non-profit organization employee; temporary or contract employee; independent business (commercial and industrial services); small office/home office; agricultural, forestry, and fishing industries; professional occupation (lawyer, tax accountant, medical-related); and other. We included prefecture of residence as a community-level variable.

### 2.3. Statistical analysis

We applied multilevel logistic regression to estimate the odds ratios (ORs) for associations of each personal infection prevention behavior and usage of Go To Travel campaign nested in the prefecture of residence. This analysis was conducted for each of the seven personal infection prevention behaviors: “wearing a mask in the presence of others;” “disinfecting hands with alcohol before going indoors;” “washing hands after using the toilet;” “gargling when returning home;” “opening windows to ventilate the room;” “carrying alcohol disinfectant;” and “disinfecting hands and washing hands after touching things.” The multivariate model was adjusted for age, sex, annual household income, educational background, marital status, and occupation as model 1; it was additionally adjusted for fear of COVID-19 infection as model 2. All covariates were treated as categorical variables. To test for trend, *p* values were calculated by treating personal infection prevention behavior as a continuous variable. We calculated the *P* values, and considered *P* values <0.05 statistically significant. All analyses were conducted with Stata (Stata Statistical Software, IC17.0; StataCorp LLC, College Station, TX, USA).

## 3. Results

Of the 605,381 people who were emailed invitations to participate, 55,045 registered monitors consented to participate in this study. Completion rate (ratio of users who finished the survey / users who agreed to participate) of this survey was 81.7%. Among them, 26,637 workers matched the subjects of this study after excluding 6,266 who gave inadequate answers and 399 who had been infected with COVID-19 and/or those who had been identified as having been in close contact with infected people.

The characteristics of the participants appear in Table 1. Among the 26,637 participants, those in their 50's were the most (33.4%), followed by those in their 40's (29.7%). Men and women were almost equally represented, with 48.9% of the respondents being women. In terms of occupation, general employees were the most common (46.5%), followed by temporary/contract employee (10.8%) and public employee, faculty member, or non-profit organization employee (10.4%).

**Table 1:** Characteristics of subjects in this study.

	***N* (%)**
Total	26,637
**Age**	
20–29	1,850 (6.9%)
30–39	4,762 (17.9%)
40–49	7,904 (29.7%)
50–59	8,909 (33.4%)
60–65	3212 (12.1%)
**Sex**	
Women	13,037 (48.9%)
**Annual household income (million JPY)**	
< 2	1,685 (6.3%)
≥2 and < 4	5,487 (20.6%)
≧4 and < 6	6,395 (24.0%)
≧6 and < 8	5,364 (20.1%)
≧8 and < 10	3,546 (13.3%)
≧10	4,160 (15.6%)
**Educational background**	
Junior high or high school	7,240 (27.2%)
Vocational school, junior college or technical school	6,441 (24.2%)
University or graduate school	12,956 (48.6%)
**Marital status**	
Married	14,794 (55.5%)
Widowed/divorced	2,809 (10.5%)
Never married	9,034 (33.9%)
**Occupation**	
General employee	12,374 (46.5%)
Manager	2,494 (9.4%)
Executive manager	850 (3.2%)
Public employee, faculty member, or non-profit organization employee	2,770 (10.4%)
Temporary/contract employee	2,871 (10.8%)
Independent business (commercial and industrial services)	2,202 (8.3%)
Small office/home office	377 (1.4%)
Agricultural, forestry, and fishing industries	212 (0.8%)
Professional occupation (lawyer, tax accountant, medical-related, etc.)	1,804 (6.8%)
Other occupation	683 (2.6%)
Fear of COVID-19 infection	19,726 (74.1%)

Table 2 presents the number and percentage of respondents implementing the seven personal infection prevention behaviors. For “wearing a mask in the presence of others” and “washing hands after using the toilet,” the majority of respondents answered “almost always: 86.5 and 85.6% respectively. Action “carrying alcohol disinfectant” was the least implemented, with 36.9% of respondents saying “almost never.”

**Table 2:** Number and percentage of respondents implementing the seven personal infection prevention behaviors.

	**Almost never**	**Not often**	**Often**	**Almost always**
(1) Wearing a mask in the presence of others	210 (0.8%)	428 (1.6%)	2,969 (11.1%)	23,030 (86.5%)
(2) Disinfecting hands with alcohol before going indoors	845 (3.2%)	2,839 (10.7%)	8,139 (30.6%)	14,814 (55.6%)
(3) Washing hands after using the toilet	238 (0.9%)	658 (2.5%)	2,927 (11.0%)	22,814 (85.6%)
(4) Gargling when returning home	3,377 (12.7%)	4,954 (18.6%)	4,711 (17.7%)	13,595 (51.0%)
(5) Opening windows to ventilate the room	1,488 (5.6%)	5,174 (19.4%)	7,995 (30.0%)	11,980 (45.0%)
(6) Carrying alcohol disinfectant	9,833 (36.9%)	4,920 (18.5%)	3,643 (13.7%)	8,241 (30.9%)
(7) Disinfecting hands and washing hands after touching things	3,396 (12.7%)	5,917 (22.2%)	7,447 (28.0%)	9,877 (37.1%)

Table 3 shows the association between each infection prevention behavior and using Go To Travel. Two of the seven preventive behaviors (“disinfecting hands with alcohol before going indoors” and “carrying alcohol disinfectant”) were positively related to using Go To Travel, that is, the more of these actions they took, the more they used Go To Travel (*p* for trend <0.001). For all results, the odds ratios were increased by additionally adding fear of COVID-19 infection to the covariates.

**Table 3:** The odds ratios and 95% confidence intervals for each personal infection prevention behaviors associated with use of Go To Travel campaign.

	**Use of Go To Travel campaign**		**Model 1**			**Model 2**		
	***n***	**%**	**OR**	**95%CI**	***p*** **value**	**OR**	**95%CI**	***p*** **value**
(1) Wearing a mask in the presence of others					0.936^†^			0.295^†^
Almost never	42	20	Reference			Reference		
Not often	109	25	1.26	0.84–1.91	0.268	1.28	0.85–1.94	0.237
Often	876	30	1.49	1.04–2.13	0.028	1.57	1.10–2.24	0.014
Almost always	6,932	30	1.37	0.97–1.94	0.077	1.48	1.05–2.11	0.027
(2) Disinfecting hands with alcohol before going indoors					0.036^†^			0.003^†^
Almost never	176	21	Reference			Reference		
Not often	774	27	1.32	1.09–1.60	0.004	1.35	1.12–1.63	0.002
Often	2,466	30	1.45	1.21–1.73	< 0.001	1.52	1.27–1.81	< 0.001
Almost always	4,543	31	1.39	1.16–1.65	< 0.001	1.47	1.23–1.75	< 0.001
(3) Washing hands after using the toilet					0.907^†^			0.387^†^
Almost never	57	24	Reference			Reference		
Not often	179	27	1.17	0.83–1.67	0.374	1.19	0.84–1.70	0.321
Often	860	29	1.24	0.91–1.71	0.174	1.30	0.95–1.78	0.106
Almost always	6,863	30	1.19	0.88–1.62	0.265	1.27	0.93–1.72	0.131
(4) Gargling when returning home					0.001^†^			0.004^†^
Almost never	1,021	30	Reference			Reference		
Not often	1,501	30	1.00	0.91–1.10	0.977	1.01	0.91–1.11	0.890
Often	1,394	30	0.96	0.87–1.06	0.471	0.97	0.88–1.07	0.578
Almost always	4,043	30	0.90	0.83–0.98	0.014	0.91	0.84–0.99	0.035
(5) Opening windows to ventilate the room					0.074^†^			0.154^†^
Almost never	416	28	Reference			Reference		
Not often	1,511	29	0.99	0.87–1.13	0.917	1.01	0.88–1.15	0.916
Often	2,436	30	1.02	0.90–1.15	0.790	1.04	0.91–1.18	0.559
Almost always	3,596	30	0.94	0.83–1.06	0.329	0.96	0.85–1.09	0.537
(6) Carrying alcohol disinfectant					0.007^†^			0.002^†^
Almost never	2,733	28	Reference			Reference		
Not often	1,439	29	1.03	0.95–1.12	0.428	1.04	0.96–1.12	0.343
Often	1,155	32	1.12	1.03–1.22	0.008	1.13	1.04–1.23	0.005
Almost always	2,632	32	1.09	1.01–1.16	0.017	1.10	1.03–1.18	0.006
(7) Disinfecting hands and washing hands after touching things					0.728^†^			0.739^†^
Almost never	888	26	Reference			Reference		
Not often	1,757	30	1.12	1.02–1.24	0.019	1.15	1.04–1.27	0.005
Often	2,328	31	1.15	1.05–1.26	0.004	1.19	1.08–1.30	< 0.001
Almost always	2,986	30	1.04	0.95–1.14	0.443	1.07	0.98–1.18	0.136

^†^p for trend.

OR, odds ratios; Cl, confidence interval.

A multilevel logistic model was used nested in prefectures of residence.

Model 1 was adjusted for age, sex, annual household income, educational background, marital status, and occupation.

Model 2 was adjusted for Model 1 and additionally fear of COVID-19 infection.

## 4. Discussion

This study found that two of the seven preventive behaviors (“disinfecting hands with alcohol before going indoors” and “carrying alcohol disinfectant”) were positively related to using Go To Travel. A dose-response relationship was observed, with the more firmly these preventive behaviors were taken, the more Go To Travel was used. For all results, the odds ratios were increased by additionally adding fear of COVID-19 infection to the covariates.

The two measures that did have a significant relationship (“disinfecting hands with alcohol before going indoors” and “carrying alcohol disinfectant”) were ones that were specifically recommended during COVID-19. The majority of respondents answered that they always practice the two habits (86.5% as “wearing a mask in the presence of others” and 85.6% as “washing hands after using the toilet”), suggesting that they have become commonplace habits. Therefore, they would not have been found to be associated with travel. It is not clear why we did not find an association in the following two actions: “opening windows to ventilate the room” and “disinfecting hands and washing hands after touching things.” It may have been difficult to recall how often either question was asked. The results of this analysis showed that the more people gargled when they returned home, the less they traveled. Gargling is not included in the preventive actions recommended by the government for COVID-19 infection control. Those who engage in the behavior of gargling may be more likely to engage in more inhibitory behaviors against infection.

Restricting travel is one of the most important public health measures a country can take to control an infectious disease pandemic(26, 27). On the other hand, if the infection pandemic continues for a long period, it will be necessary to simultaneously implement measures to stimulate the economy in response to the infection situation. The Go To Travel program implemented in Japan was one of the measures to stimulate the economy. People who normally engage in infection prevention behaviors are more sensitive to the risk of infection and may refrain from traveling due to anxiety about infection. In this study, the data demonstrated that even after taking into account the impact of fear of COVID-19 infection, those who were taking infection prevention behaviors were still using Go To Travel. The fact that people with good infection prevention behavior tend to travel more is a desirable public health situation.

Before Go To Travel was launched, a nationwide state of emergency was declared in Japan in April 2020 (2). In accordance with that, citizens were asked to refrain from going out after 8 p.m., abstain from going out during the daytime for unnecessary reasons, and refrain from traveling across prefectural borders as much as possible (28). Those restrictions were eased after the state of emergency was lifted. However, to counter a reemergence of infection, cooperation is still required to continue measures. Thus, people who followed infection prevention behaviors—especially those recommended for COVID-19—were more likely to use Go To Travel; this finding suggests that many Go To Travel users may have been taking appropriate infection control measures when traveling. However, many Go To Travel users adopted inadequate infection control behaviors; accordingly, it is necessary to continue strict application of infection prevention behaviors at travel destinations.

Greater confidence in governmental programs has been reported to be positively correlated with infection prevention behaviors (15). In that regard, high perceived severity and perceived susceptibility indicate a high estimate of risk for infectious disease; therefore, those factors may inhibit travel and other outdoor behavior. Conversely, perceived self-efficacy and effectiveness may lead to an awareness of the effectiveness of infection prevention behaviors when traveling; they may have a positive effect on traveling. Confidence in the government is also considered positive toward accepting the government-led Go To Travel campaign. Among factors that facilitate infection prevention behaviors, there are both ones that inhibit and promote using Go To Travel. The relationship between infection prevention and outside behaviors is likely influenced by the balance between those potentially positive and negative factors. Go to Travel was implemented in a relatively stable infection situation; thus, that balance may have resulted in a positive relationship.

This study is the first to determine the relationship between individual infection prevention behavior and the use of government-sponsored travel promotion campaigns (Go To Travel). Travel behavior is discouraged in situations where the pandemic has not fully ended (26). However, when the infection situation has calmed down, measures to facilitate travel with appropriate infection prevention measures will be necessary. The government needs to understand that the use of public transportation and other means of transportation is declining during the COVID-19 pandemic and counteract it with COVID-19-specific policy measures that will revive a sense of safe and secure travel among the public. Furthermore, it is assumed that vulnerable groups such as the sick and disabled will behave more inhibitively when moving during a pandemic, but it is also noted that there are no studies specifically on these individuals (29). Further research is needed on what measures are needed to ensure that vulnerable groups are able to move around safely and securely.

The present study has a couple of limitations. First, the questions related to infection prevention behaviors in our survey were not limited to using Go To Travel; there were also questions regarding such behaviors in daily life. Our questionnaire was not restricted to activities at destinations; thus, the participants may have responded with respect to their activities at home, not at tourist destinations. However, it is unlikely that a person who does not normally adopt preventive actions will behave appropriately only when traveling. Second, the generalizability of the results should be applied with caution because our survey was conducted using an Internet panel. However, the random sampling, stratified by gender, age, and region, helped ensure some degree of representativeness of the results. Third, we could not ascertain the response rate because of the Internet survey. Selection bias is unavoidable in Internet surveys because respondents are not representative of any group (30, 31). To minimize selection bias as much as possible, we stratified our sampling by sex, office workers/non-office workers, and region by cumulative COVID-19 incidence rate. By measuring various health outcome measures, we confirmed that there is no significant bias compared to the general population in Japan (17).

## 5. Conclusions

In this study, we observed two of the seven preventive behaviors (“disinfecting hands with alcohol before going indoors” and “carrying alcohol disinfectant”) were positively related to using Go To Travel. To balance pandemic preparedness with economic preparedness, it is also necessary to promote travel when the infection situation is calm. However, since human mobility can be a factor that exacerbates the infection situation, it is necessary to promote more infection prevention behaviors among individuals. We confirmed that Go To Travel users were basically doing a good infection prevention behaviors, but they tended not to wash their hands after touching things or carry alcohol sanitizer. It is necessary to encourage these measures to be taken when traveling.

## Data availability statement

The raw data supporting the conclusions of this article will be made available by the authors, without undue reservation.

## Ethics statement

The studies involving human participants were reviewed and approved by the Ethics Committee of the University of Occupational and Environmental Health, Japan (Reference Nos. R2-079 and R3-006). Written informed consent for participation was not required for this study in accordance with the national legislation and the institutional requirements.

## Author contributions

HS: analysis and writing the manuscript. TN: creating the questionnaire, analysis, and drafting the manuscript. TI, AH, HA, KMu, ST, and MT: review of manuscripts, interpretation, and funding for research. KMo: drafting and review of manuscripts and interpretation. All authors have read and approved the final manuscript.
